# Sonography Findings Predict Testicular Viability in Pediatric Patients With Testicular Torsion

**DOI:** 10.7759/cureus.21790

**Published:** 2022-01-31

**Authors:** Lisa B Shields, Michael W Daniels, Dennis S Peppas, Eran Rosenberg

**Affiliations:** 1 Neurological Surgery, Norton Healthcare, Norton Neuroscience Institute, Louisville, USA; 2 Bioinformatics and Biostatistics, University of Louisville, Louisville, USA; 3 Pediatric Urology, Norton Healthcare, Louisville, USA

**Keywords:** orchiectomy, ultrasound, testicular torsion, pediatric surgery, pediatric urology

## Abstract

Background: Testicular torsion poses a pediatric surgical emergency that necessitates rapid diagnosis and surgery to prevent testicular loss. We sought to determine whether any particular findings on Doppler ultrasound (US) were predictive of testicular viability in pediatric patients with testicular torsion.

Materials and methods: We identified males between ages one and 18 years who experienced testicular torsion over a six-year period (January 1, 2015-December 31, 2020). All patients were evaluated at our institution’s emergency department by a pediatric urologist and underwent a Doppler scrotal US.

Results: Of the 140 patients with testicular torsion, 56 (40%) had a non-viable testis and underwent an orchiectomy, while 84 (60%) had a viable testis and orchiopexy. Testicular heterogeneity (47 [84%] vs 48 [57%], p = 0.001), epididymis heterogeneity (23 [41%] vs 21 [25%], p = 0.063), and scrotal wall thickening (25 [45%] vs 5 [6%], p < 0.001) were significantly associated with a non-viable testis. Epididymis heterogeneity (adj. odds ratio [OR] = 0.33 [0.13, 0.79], p = 0.013) and scrotal wall thickening (adj. OR = 0.08 [0.03, 0.24], p < 0.001) exhibited significantly lower odds for viability. Testicular heterogeneity and scrotal wall thickening were more likely to develop with a longer duration of symptoms (both p < 0.001).

Conclusion: Our study determined that certain Doppler scrotal US findings, specifically, testicular and epididymal heterogeneity as well as a thickened scrotal wall, are associated with testicular demise in patients with testicular torsion. As testicular heterogeneity and scrotal wall thickening are more likely to arise with a longer symptom duration, an urgent diagnosis and prompt surgical intervention are imperative to avert testicular loss.

## Introduction

With an incidence of 1/1500 or 3.8% of males < 18 years, testicular torsion may develop when the spermatic cord rotates on its own axis resulting in decreased or absent blood flow to the testes [1-5]. Accounting for 20%-25% of acute pediatric scrotal cases, testicular torsion may lead to ischemic testicular damage if not promptly recognized and surgically treated [1,2,6,7]. Surgery within six hours of pain onset boasts a testicular salvage rate of >90%, which plunges to 50% after 12 hours and <10% after 24 hours with complete testicular infarction [1,2,4,8,9]. In this respect, the likelihood of testicular salvage is directly related to the time between symptom onset and surgery.

Several clinical findings are closely associated with testicular torsions such as the sudden onset of severe testicular and spermatic cord pain, lower abdominal pain, nausea, vomiting, testicular swelling, abnormal cremasteric reflex, horizontal testicular lie, and high testicular position [1-4,6,8,10-12]. While obtaining a history of testicular torsion is valuable in the detection of testicular torsion, diagnosing this condition based solely on the clinical history may be difficult as 50%-86% of testicular explorations result in negative findings despite numerous clinical scoring systems [13].

Doppler ultrasound (US) serves as a valuable tool to increase the predictability of testicular torsion and to differentiate testicular pathologies that may mimic torsion. The grayscale US reveals an alteration in vascular flow course, whirlpool sign in the spermatic cord, changes in testicular echogenicity and size, presence of a hydrocele, and scrotal wall thickening [2]. With a sensitivity of 89%-100% and a specificity of 69%-99% [1-4,8,9,14], the color Doppler US accurately shows the size, shape, echogenicity, and perfusion of the testes [4,8,15]. Positive features of US include its ready availability, easy portability, high accuracy, noninvasive, lack of ionizing radiation or sedation, and low costs; however, it is also highly dependent on the operator, may be associated with a time delay to surgery, and may convey the false-negative findings where surgery would not be pursued [3,4,6,8,10,16]. The combination of clinical findings and the US has been shown to reduce the rate of negative exploration by 10% without increasing the rate of missed testicular torsion [4,5]. Furthermore, it has been reported that the US may prevent unnecessary scrotal explorations in patients with acute scrotal pain [17].

The present study investigated whether specific findings on the US are more likely to be predictive of testicular viability in pediatric patients with testicular torsion. Morphological changes of the testes, epididymis, and spermatic cord in the setting of testicular torsion are presented. The differential diagnostic pathologies of acute scrotal pain and their distinguishing US characteristics are also discussed.

## Materials and methods

Under an Institutional Review Board-approved protocol and conforming to the World Medical Association Declaration of Helsinki, we identified male children and adolescents of ages one to 18 years who experienced testicular torsion over a six-year period (January 1, 2015-December 31, 2020), with special attention devoted to the Doppler scrotal ultrasound (US) findings. All patients were evaluated in our institution’s emergency department (ED) by a pediatric urologist who obtained the medical history and performed a focused genitourinary physical examination. A pediatric urologist was available at all times to evaluate and treat patients with testicular torsion. Four pediatric urologists are employed at our institution, and the on-call schedule alternates between all four. All patients underwent a Doppler US that was performed by a pediatric radiologist. As the US is adjacent to the ED, the time to complete the US was very short.

Numerous metrics observed on the US were obtained including testicular volume, difference in testicular volume between sides, testicular heterogeneity, presence of blood flow on the affected side of the testis and epididymis, enlarged and heterogeneous epididymis, thickened scrotal wall, hydrocele, varicocele, calcifications, necrosis, and hemorrhage. Additional metrics comprised the patient’s age, duration of symptoms, and type of surgery (orchiectomy vs. orchiopexy).

The University of Louisville Institutional Review Board (IRB Number: 20.0778) determined that our study was exempt according to 45 CFR 46.101(b) under Category 4. The parents of the patients with testicular torsion provided written informed consent.

Statistical analysis

Group comparisons between viable (orchiopexy) to non-viable (orchiectomy) testes were evaluated with the Fisher’s exact test for categorical variables (count, %) and Wilcoxon rank-sum test for ordinal variables (median (IQR]). The Kruskal-Wallis test on ranks examined group differences across symptom duration time periods. Backward feature selection determined the best fit model from significant univariate characteristics of patients with testicular torsion based on the Akaike information criterion (AIC). Multivariable results summarized adjusted odds ratios (OR) with 95% confidence intervals from the best fit model. All analyses were performed using R software version 4.0.3 [18].

## Results

Univariate analysis

Of the 140 patients with testicular torsion over the six-year duration of this study, 56 (40%) had a non-viable testis and underwent an orchiectomy, while 84 (60%) had a viable testis and orchiopexy. Three variables observed on the US were significantly associated with a non-viable testis by univariate analysis, including testicular heterogeneity (47 [84%] vs 48 [57%], p = 0.001), epididymis heterogeneity (23 [41%] vs 21 [25%], p = 0.063), and thickened scrotal wall (25 [45%] vs 5 [6%], p < 0.001) (Table 1).

**Table 1:** Features comparing a viable testis versus a non-viable testis for pediatric males at our institution (January 1, 2015-December 31, 2020)

Characteristics	Overall N = 140	Orchiectomy N = 56	Orchiopexy N = 84	P-value
Testicular heterogeneity = Yes	95 (68)	47 (84)	48 (57)	0.001
Presence of blood flow to affected testis = Yes	4 (3)	1 (2)	3 (4)	0.650
Testicular volume (Left)	11.1 (7.4, 15.0)	10.5 (7.3, 15.0)	11.6 (7.6, 15.5)	0.243
Testicular volume (Right)	11.8 (7.9, 16.0)	10.3 (4.6, 14.8)	12.7 (9.5, 16.9)	0.024
Difference in testicular volumes	1.5 (1.2, 1.9)	1.5 (1.2, 2.0)	1.4 (1.1, 1.7)	0.097
Enlarged epididymis = Yes	67 (48)	29 (52)	38 (45)	0.492
Heterogeneous epididymis = Yes	44 (31)	23 (41)	21 (25)	0.063
Blood flow to epididymis = Yes	107 (76)	39 (70)	68 (81)	0.155
Thickened scrotal wall = Yes	30 (21)	25 (45)	5 (6)	<0.001
Hydrocele = Yes	90 (64)	28 (50)	62 (74)	0.007
Varicocele = Yes	5 (4)	2 (4)	3 (4)	1.000
Testicular calcifications = Yes	2 (1)	2 (4)	0 (0)	0.158
Testicular necrosis = No	140 (100)	56 (100)	84 (100)	NA
Testicular hemorrhage = Yes	2 (1)	2 (4)	0 (0)	0.158

**Figure 1:**
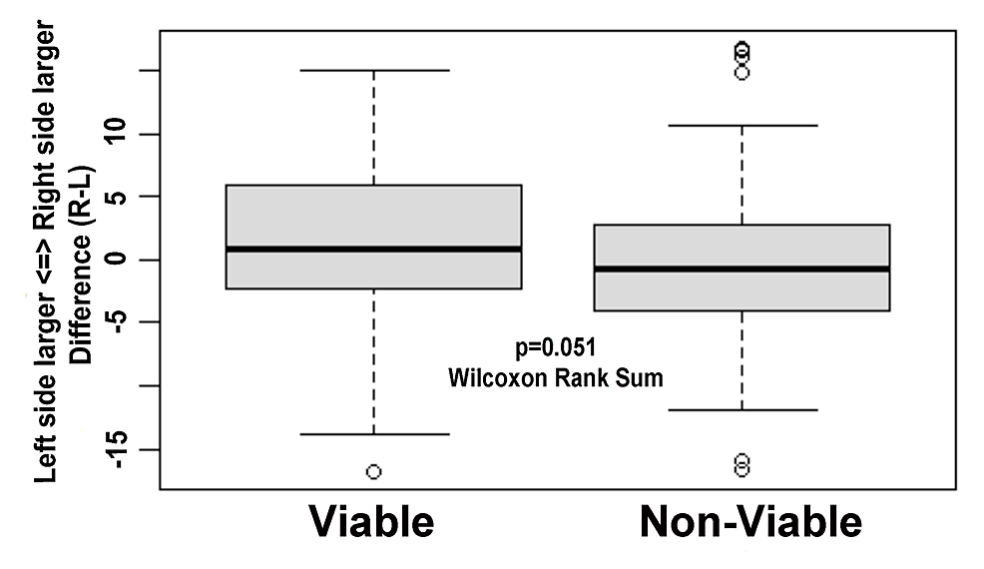
Difference in testicular volume between the left and right testes comparing the viable and non-viable testes A true difference (not absolute) in size (right minus left) revealed significantly more left-sided differences in testicular volume in non-viable testis outcomes (p = 0.05).

A true difference (not absolute) in size (right minus left) revealed significantly (p = 0.05, Wilcoxon rank-sum test) more left-sided differences in testicular volume in non-viable testis outcomes (Figure 1).

**Figure 2:**
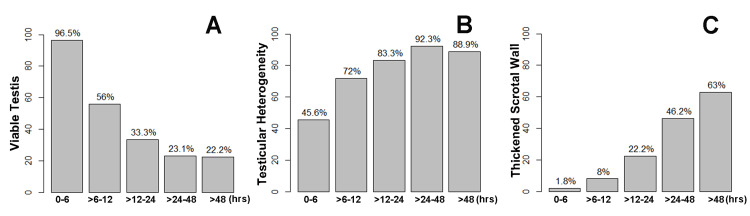
Testicular viability, testicular heterogeneity, and thickened scrotal wall in relation to symptom duration of testicular torsion (A) Pediatric patients with testicular torsion who had a viable testis and underwent an orchiopexy were more likely to have a symptom duration < six hours (p < 0.001). (B) Testicular heterogeneity and (C) a thickened scrotal wall on preoperative ultrasound were more likely to develop with a longer duration of symptoms (both p < 0.001).

When stratified by five levels of symptom duration, older patients and those who had a viable testis and underwent an orchiopexy were more likely to have a symptom duration < six hours (p = 0.001 and p < 0.001, respectively) (Figure 2, Panel A; Table 2).

**Table 2:** Features stratified by duration of testicular torsion symptoms for pediatric males at our institution (January 1, 2015-December 31, 2020)

Characteristics	Overall N = 140	0-6 hours N = 57	>6-12 hours N = 25	>12-24 hours N = 18	>24-48 hours N = 13	>48 hours N = 27	P-value
Age at the time of surgery	14.0 (12.7, 15.3)	14.6 (13.8, 15.6)	13.8 (12.6, 14.8)	12.8 (11.2, 13.8)	13.4 (7.6, 14.1)	13.8 (12.3, 15.2)	0.001
Testicular heterogeneity = Yes	95 (68)	26 (46)	18 (72)	15 (83)	12 (92)	24 (89)	<0.001
Presence of blood flow to affected testis = Yes	4 (3)	3 (5)	0 (0)	1 (6)	0 (0)	0 (0)	0.615
Testicular volume (Left)	11.1 (7.4, 15.0)	12.3 (10.1, 14.8)	10.9 (6.8, 14.5)	10.2 (5.3, 16.0)	8.4 (2.3, 11.7)	10.6 (7.4, 16.4)	0.433
Testicular volume (Right)	11.8 (7.9, 16.0)	12.1 (9.9, 16.1)	13.3 (6.5, 20.4)	10.0 (6.0, 14.4)	8.2 (1.8, 14.1)	11.9 (7.9, 15.9)	0.104
Difference in testicular volumes	1.5 (1.2, 1.9)	1.3 (1.1, 1.6)	1.5 (1.3, 1.9)	1.8 (1.4, 2.7)	1.9 (1.2, 2.2)	1.5 (1.1, 1.9)	0.012
Enlarged epididymis = Yes	67 (48)	25 (44)	14 (56)	9 (50)	8 (62)	11 (41)	0.637
Heterogeneous epididymis = Yes	44 (31)	15 (26)	9 (36)	7 (39)	8 (62)	5 (19)	0.069
Blood flow to epididymis = Yes	107 (76)	47 (82)	18 (72)	13 (72)	8 (62)	21 (78)	0.478
Thickened scrotal wall = Yes	30 (21)	1 (2)	2 (8)	4 (22)	6 (46)	17 (63)	<0.001
Hydrocele = Yes	90 (64)	44 (77)	19 (76)	9 (50)	6 (46)	12 (44)	0.008
Varicocele = Yes	5 (4)	3 (5)	0 (0)	1 (6)	0 (0)	1 (4)	0.906
Testicular calcifications = Yes	2 (1)	0 (0)	1 (4)	0 (0)	1 (8)	0 (0)	0.112
Testicular necrosis = No	140 (100)	57 (100)	25 (100)	18 (100)	13 (100)	27 (100)	NA
Testicular hemorrhage = Yes	2 (1)	0 (0)	0 (0)	0 (0)	1 (8)	1 (4)	0.184

Testicular heterogeneity and scrotal wall thickening were more likely to develop with a longer duration of symptoms (both p < 0.001) (Figure 2, Panels B and C; Table 2). The difference in testicular volume between the left and right testes and epididymal heterogeneity also exhibited statistical significance when stratified by five levels of symptom duration (p = 0.012 and p = 0.069, respectively).

Multivariable regression

In order to predict testicular viability from more than one factor, six univariate-associated variables were simultaneously evaluated as candidates for the best fit final logistic model. Fixing age and difference in testicular volume (%), the backward selection removed testicular heterogeneity with three characteristic variables remaining. Three variables exhibited lower odds for viability: testicular volume difference (%) (adj. OR = 0.99 [0.97, 1.01], p = 0.362), epididymis heterogeneity (adj. OR = 0.33 [0.13, 0.79], p = 0.013), and scrotal wall thickening (adj. OR = 0.08 [0.03, 0.24], p < 0.001) (Table 3).

**Table 3:** Odds ratio for testicular viability in pediatric males at our institution (January 1, 2015-December 31, 2020)

Characteristics	Adj. OR	Lower CI	Upper CI	P-value
(Intercept)	0.41	0.06	3.05	0.385
Age	1.16	1	1.33	0.043
Difference in testicular volumes	0.99	0.97	1.01	0.362
Heterogeneous epididymis	0.33	0.13	0.79	0.013
Thickened scrotal wall = Yes	0.08	0.03	0.24	0
Hydrocele = Yes	2.66	1.07	6.57	0.034

The other two variables in the final model exhibited higher odds for viability: age (adj. OR = 1.16 [1.00, 1.33], p = 0.043) and hydrocele (adj. OR = 2.66 [1.07, 6.57], p = 0.034).

## Discussion

While US is the best modality for detecting testicular torsion, diagnosing intermittent (also known as torsion-detorsion), incomplete/partial, and complete testicular torsion may prove challenging [8,19]. As ischemia is a progressive process, the US findings may change over time. With intermittent torsion there is a sudden onset of unilateral testicular pain for a short duration which spontaneously resolves while partial or incomplete torsion involves less than a 360-degree twisting of the spermatic cord with some residual perfusion to the testis [1,10,14,16,19]. Even with complete testicular torsion where the degree of twisting is 360 degrees or greater usually resulting in absent testicular blood flow, situations may arise when the flow is preserved or decreased [19]. Therefore, the presence of intratesticular flow does not exclude testicular torsion, and scrotal exploration should not be delayed if the medical history (characteristic symptoms of testicular torsion and short duration of pain) and physical examination strongly suggest testicular torsion [7,19,20]. 

Several pathological findings may be observed on the US with testicular torsion involving the testes, spermatic cord, and epididymis. Morphological changes include an altered course and whirlpool or snail-shell appearance of the spermatic cord, increased testicular size, ipsilateral hydrocele development, variations in testicular echotexture, epididymal enlargement, and a thickened scrotal wall [1,14]. Testicular non-viability is associated with an enlarged and heterogeneous echotexture of the testis, reduced testicular perfusion, thickened scrotal wall, and duration of pain onset to hospital admission > six hours [1,4,8,19]. The swollen testis results from vascular congestion and edematous changes and is larger in volume compared to the asymptomatic side [2,19,21]. The affected testis often has a normal echogenicity on the US in the early stages of testicular torsion, which subsequently becomes enlarged and heterogeneous due to hemorrhage and necrosis as the torsion progresses [1,4,10,16]. Arterial and venous waveforms may be detected with a spectral Doppler, with the venous circulation compromised first in testicular torsion due to the low-pressure system [21]. If the arterial waveforms are present but the venous ones are absent, early testicular torsion or incomplete torsion may be present.

While most studies highlighting testicular torsion focus on the testicular abnormalities, the epididymis may also exhibit morphological alterations. In Afsarlar et al.’s study of 27 patients with acute testicular torsion, the mean epididymis size and twisting degree were significantly higher in torsed testes than in the contralateral epididymis (<0.001) [1]. Furthermore, there is a higher number of epididymal cystic structures associated with testicular non-viability (p = 0.025) and a higher twisting degree (p = 0.017). These authors suggest that the epididymis may serve as a potential prognostic indicator of testicular viability. A hypovascular or avascular enlarged epididymis with preserved testicular vascularity is also concerning for testicular torsion [19]. 

Myriad disorders comprise the differential diagnosis of the acute scrotum and are usually due to vascular compromise, infection, or trauma. Due to the overlapping clinical symptoms and potentially problematic physical examination due to the exquisite tenderness and increased swelling of the testes, US is the gold standard in differentiating between these conditions. The acutely torsed testicular appendage is the most common cause of acute scrotum and results from ischemia; epididymitis, orchitis, and epididymo-orchitis often have an infectious etiology, although they may be idiopathic or due to trauma or medications [6-8,16,19,22]. Unlike testicular torsion that requires prompt surgical intervention to salvage the testis, these disorders are treated medically. US findings of torsion of the testicular appendage include hyperemia surrounding the testicular appendage and preservation of normal blood flow within the testis [8]. Epididymitis is marked by an enlarged, hypoechoic, and heterogeneous epididymis as well as increased blood flow, reactive hydroceles, and scrotal wall thickening [8,10,14,21]. Other causes of acute scrotal pain that should be considered in the differential diagnosis include testicular trauma, an epididymal appendage, cellulitis, tumor, hernia, hydrocele, and varicocele [9,15,16]. 

Point-of-care US (POCUS), specifically US performed by the pediatric ED physician as opposed to the comprehensive US by radiologists, has been shown to agree with the final diagnosis in 70% of cases of the acute scrotum, with a higher percentage attained by more experienced POCUS users [9,22]. Additionally, POCUS results were received a median of 73 minutes before those from the radiology department [22].

Strengths and limitations

Our comprehensive six-year study of children and adolescents with testicular torsion highlights the importance of a preoperative US in uncovering certain findings that may be predictive of testicular demise. Our results corroborate previous studies in the literature that testicular and epididymal heterogeneity and a thickened scrotal wall are significantly more likely to be associated with a non-viable testis, thus necessitating an orchiectomy. We also determined that testicular heterogeneity and scrotal wall thickening are significantly more likely to develop with a longer duration of symptoms; therefore, a rapid diagnosis and timely surgical intervention are mandatory to prevent testicular loss. Published articles in the literature highlighting the use of US in testicular torsion primarily emphasize blood flow to the testis. Our study focused on numerous parameters in addition to testicular blood flow that predict testicular viability. A commonly reported disadvantage of obtaining a preoperative US to diagnose testicular torsion is the length of time taken in performing this procedure and receiving the results. A strength of the current study is that the US was located next to our ED; thus, the test was hastily executed with the results quickly attained. Additionally, our ED has a testalgia protocol that expedites radiological imaging in these patients. Our large number of patients with testicular torsion in a metropolitan setting who underwent the US serves as a model that may be replicated in other similar communities.

The limitations of the current study are its retrospective nature and the fact that it focused only on patients who had testicular torsion and not on all cases of acute scrotum. 

## Conclusions

The clinical presentation of testicular torsion is challenging due to the overlapping findings of testicular torsion and other acute scrotal disorders. A Doppler US provides excellent anatomical detail, which is valuable for all types of pediatric scrotal pathology. With its ability to accurately assess acute scrotum and scrotal masses, US is a beneficial imaging modality to differentiate between surgical emergencies and conditions that can be managed conservatively. As testicular and epididymal heterogeneity as well as a thickened scrotal wall are significantly associated with a non-viable testis and are time-dependent factors, obtaining an urgent US followed by expedited surgical intervention offers the best prognosis for restoring testicular blood flow and preserving the testis.
